# Supplementary material for: Hepatic expression of GAA results in enhanced enzyme bioavailability in mice and non-human primates
Source: Nat Commun. 2021 Nov 4;12:6393. doi: 10.1038/s41467-021-26744-4 (PMC8568898; doi:10.1038/s41467-021-26744-4)
Supplement: Supplementary file 1 — Supplementary Information [file 41467_2021_26744_MOESM1_ESM.pdf]

Supplementary Information

Supplementary Figures

Supplementary Figure 1

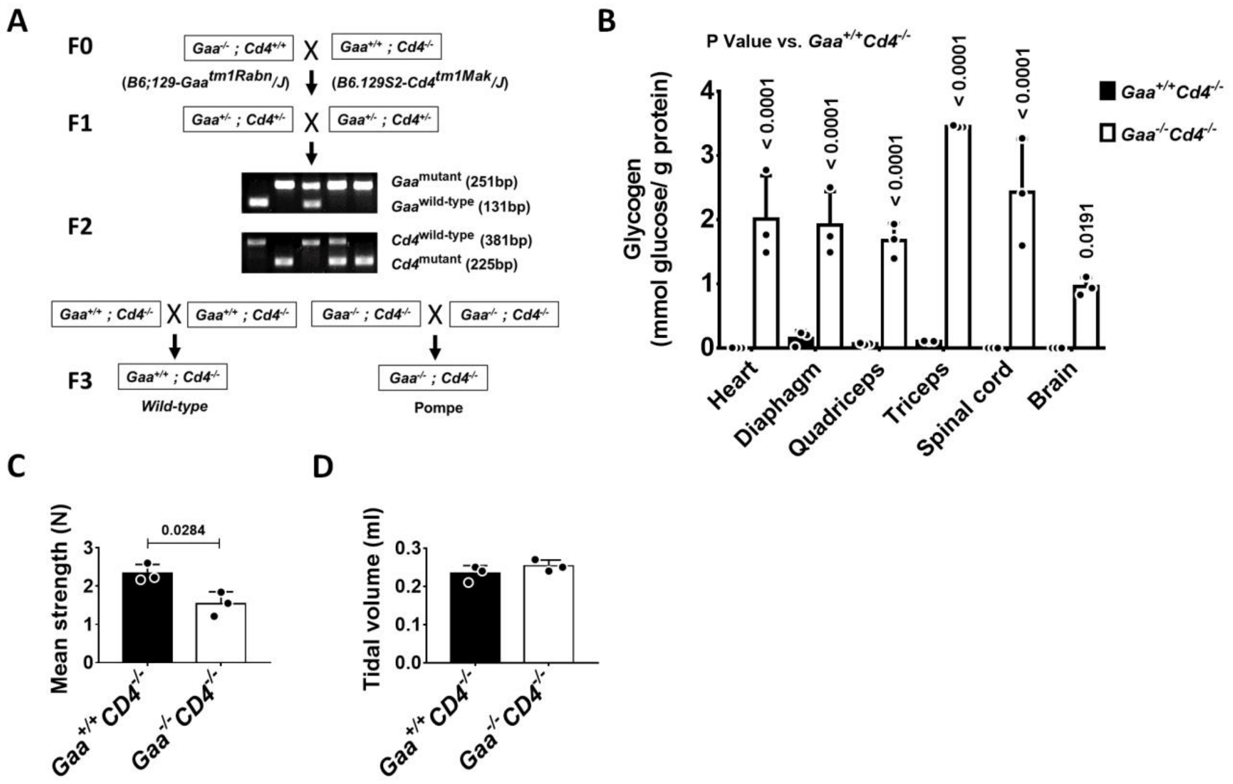

Supplementary Fig. 1. Characterization of the *Gaa*$^{-/-}$*Cd4*$^{-/-}$ mouse model. **A** Breeding scheme for the generation of the *Gaa*$^{-/-}$*Cd4*$^{-/-}$ mouse colony. Analysis of glycogen content in **B** tissues**, C** grip strength, and **D** respiratory Tidal volume in 4-month-old mice. Data shown as average ± SD. Statistical analysis: B Two-way ANOVA with Bonferroni correction; C, D Two-sided t-test ; B-D, n = 3 per group.

Supplementary Figure 2

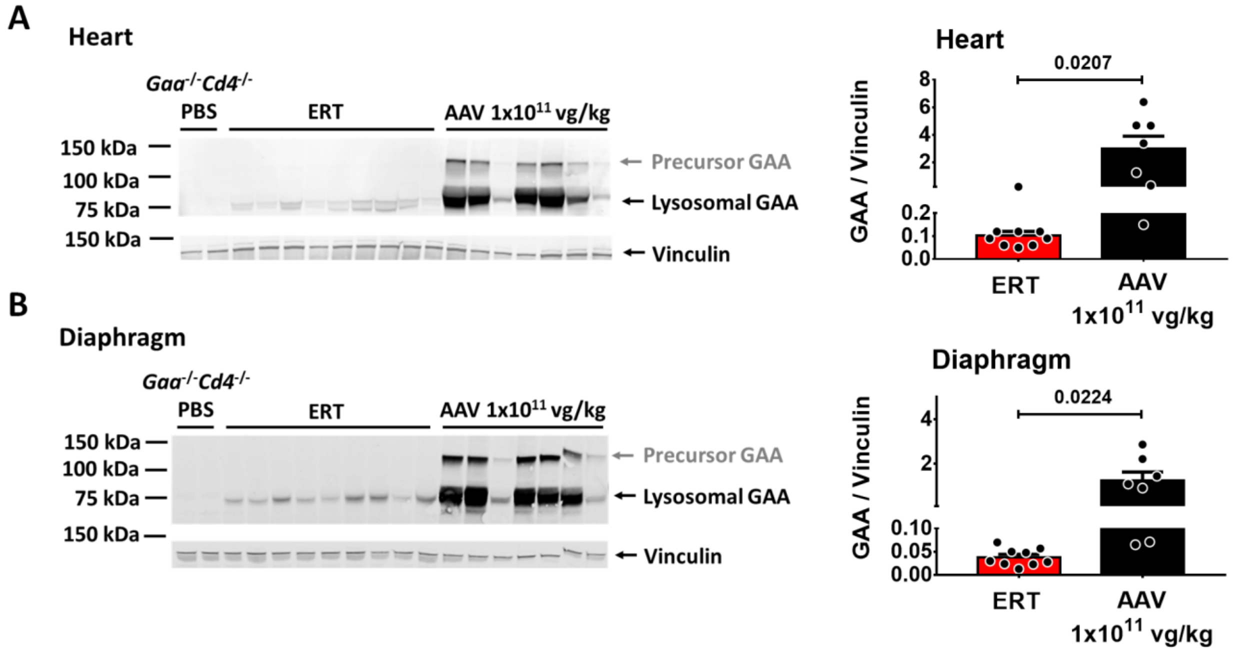

Supplementary Fig. 2. Analysis of lysosomal GAA levels in tissues of mice treated with enzyme replacement therapy (ERT, n = 9) or AAV-GAA at 1x10^{11} vg/kg (n = 7). **A, B** Western blot and quantification of lysosomal GAA levels in heart (A) and diaphragm (B). Data shown as average ± SD. Statistical analysis was performed by two-sided t-test; n = 7-9.

Supplementary Figure 3

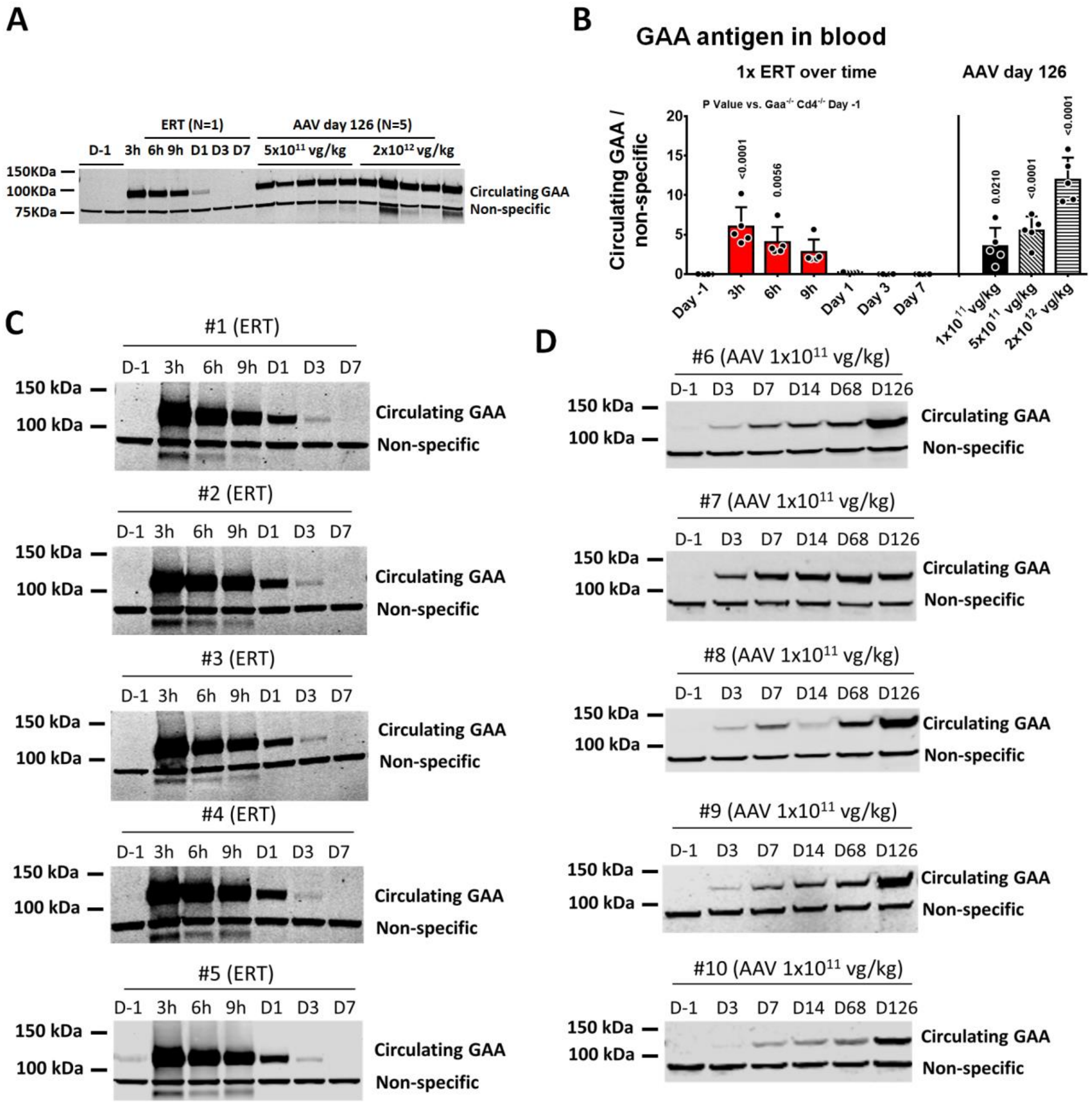

Supplementary Fig. 3. GAA kinetics in circulation. **A** Representative Western blot analysis of GAA levels in blood of mice that received a single injection of rhGAA (enzyme replacement therapy, ERT, at 20 mg/kg) or AAV-GAA at $5x10^{11}$ and $2x10^{12}$ vg/kg. ERT represents the time course of rhGAA clearance from plasma in a single mouse. AAV-GAA represents GAA levels at sacrifice (day 126) in individual mice. The results shown were reproducible in all animals tested (n = 5) and across multiple experiments. **B**

Quantification of GAA antigen levels in blood (Data shown as average ± SD, n= 5 per group). **C,D** Western blot analysis of circulating GAA enzyme over time in individual mice that received a single injection of rhGAA (ERT at 20mg/kg) (C) or AAV-GAA at $1x10^{11}$ vg/kg (D). Statistical analysis: B One-way ANOVA with Tukey post-hoc.

Supplementary Figure 4

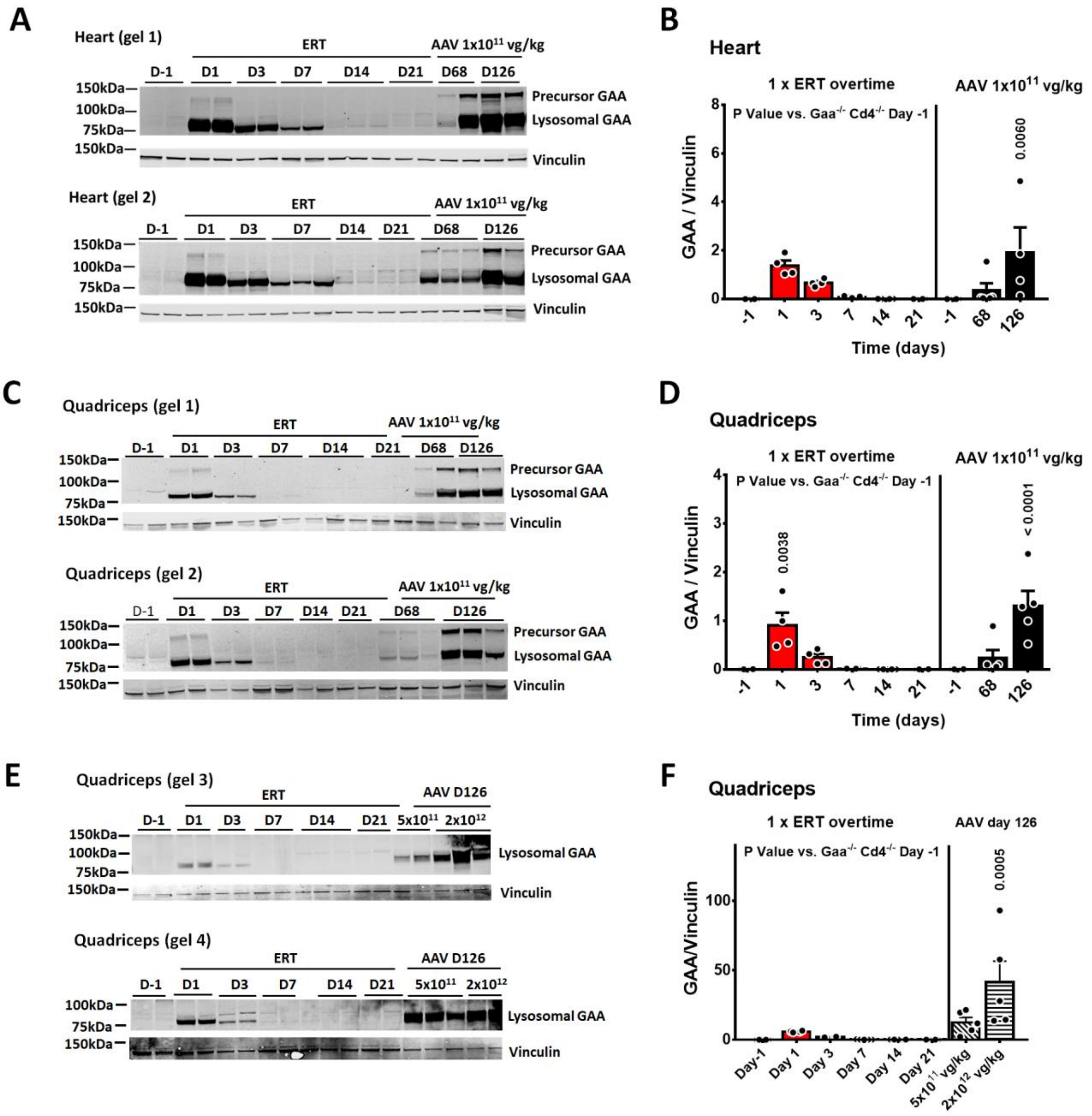

Supplementary Fig. 4. GAA enzyme kinetics in heart and quadriceps evaluated by Western blot analysis. A-F Western blot and quantification of lysosomal GAA amounts in heart (A,B) and quadriceps (C-F) of mice that received a single injection of rhGAA (ERT at 20 mg/kg) or AAV-GAA at $1x10^{11}$ (B, D), $5x10^{11}$ (E, F), or $2x10^{12}$ vg/kg (E, F). Enzyme replacement therapy (ERT). Data shown as average ± SD. Statistical analysis: One-way

ANOVA with Tukey post-hoc. Each lane in panels A,C,E represents one individual animal (n = 4 or 5 per time point). Panels B,D,F n = 4 or 5 as in panels A,C,E.

Supplementary Figure 5

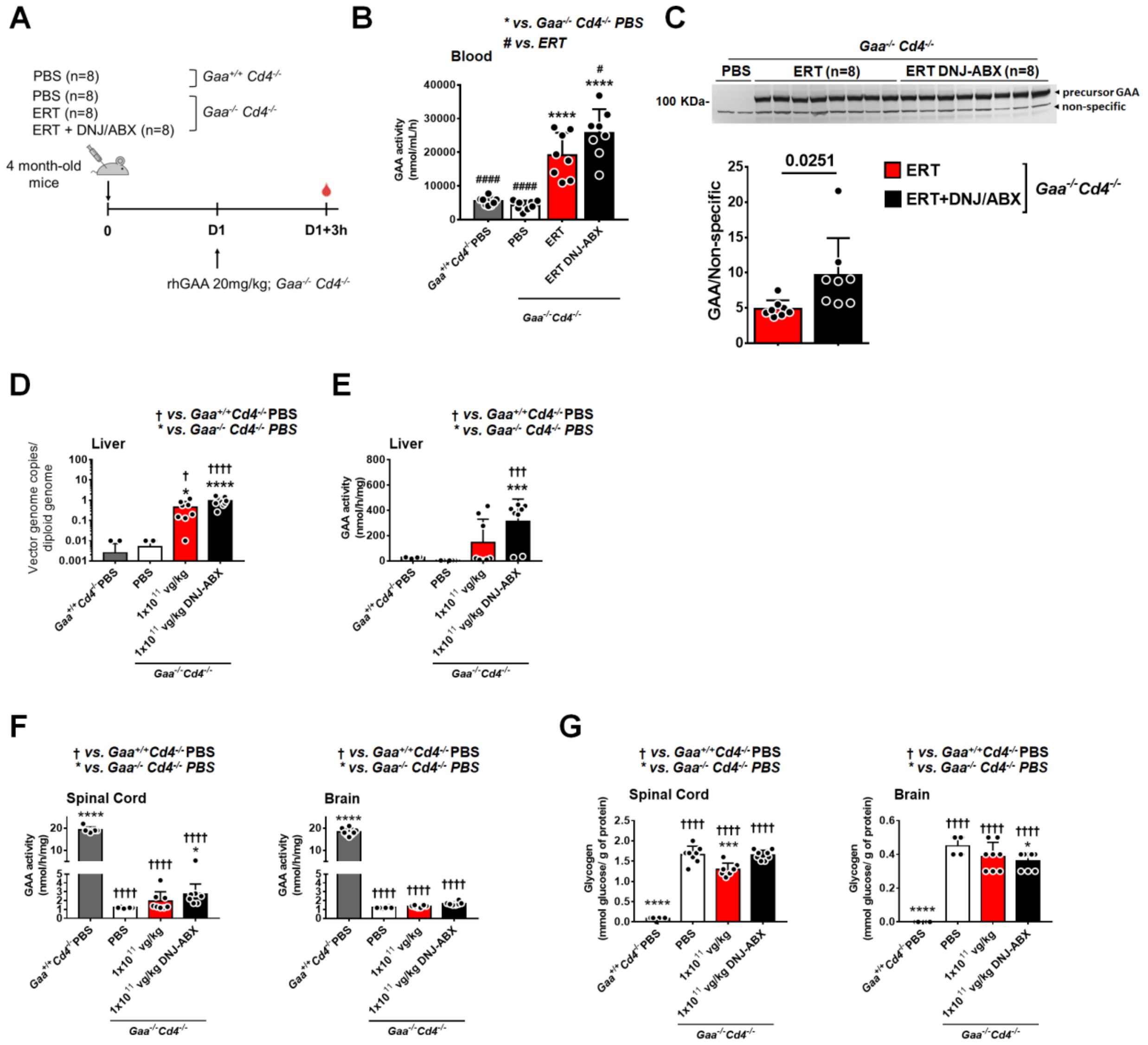

Supplementary Fig. 5. Evaluation of combined therapy with pharmacological chaperones. **A-C** Evaluation of pharmacological chaperone (PC) therapy combined with enzyme replacement therapy (ERT). **A** Experimental design: 4-month-old *Gaa*$^{-/-}$*Cd4*$^{-/-}$ male mice were infused with ERT in combination with 1-Deoxynojirimycin (DNJ) and ambroxol (ABX). Untreated *Gaa*$^{+/+}$*Cd4*$^{-/-}$ and *Gaa*$^{-/-}$*Cd4*$^{-/-}$ mice were used as controls. **B** GAA activity measured in blood 3 hours after ERT. **C** Western blot of circulating GAA antigen levels 3 hours after ERT. **D-G** Evaluation of the combined efficacy of AAV-GAA gene therapy with PCs in 4-month-old *Gaa*$^{-/-}$*Cd4*$^{-/-}$ (see Figure 8A for experimental

design). **D** Analysis of vector genome copy number in liver at sacrifice. **E** GAA enzyme activity in liver at sacrifice. **F-G** Analysis of GAA activity (F) and glycogen content (G) in spinal cord and brain. Data shown as average ± SD. Statistical analysis: B, D, E, F, G One-way ANOVA with Tukey post-hoc; C two-sided t-test. *, † and $^{\#}$ $p<0.05$; ** $p<0.01$; *** and ††† $p<0.001$; ****, †††† and $^{\#\#\#\#}$ $p<0.0001$. Exact p values for panels B,D,E,F,G are provided in the Source Data file. B-G: n = 8 mice per treatment group.

Supplementary Figure 6

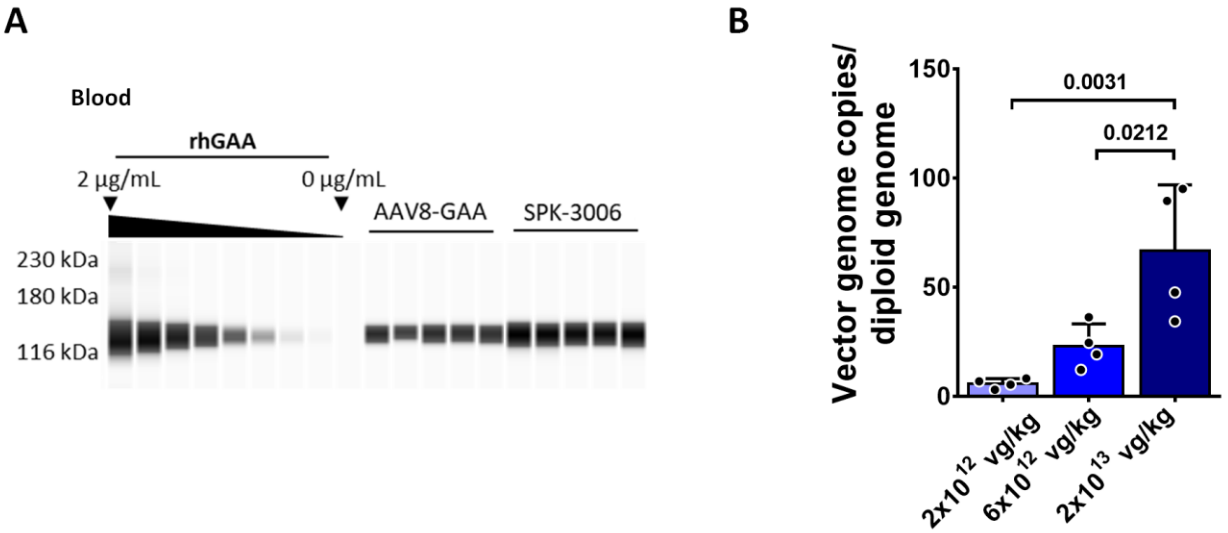

Supplementary Fig. 6. Additional evaluations of SPK-3006 in mice and non-human primates. **A** GAA antigen amounts measured in the blood of C57BL/6 mice 4 weeks after the administration of AAV-GAA or SPK-3006 vectors at $2x10^{12}$ vg/kg (n = 5 mice per group) and evaluated using capillary electrophoretic immunoassay. **B** Analysis of SPK-3006 vector genome copies per haploid genome in livers of NHPs at sacrifice (day 28 after vector administration) (data shown as average ± SD). Statistical significance: One-way ANOVA with Tukey post-hoc, n = 4 NHPs per group.

Supplementary Figure 7

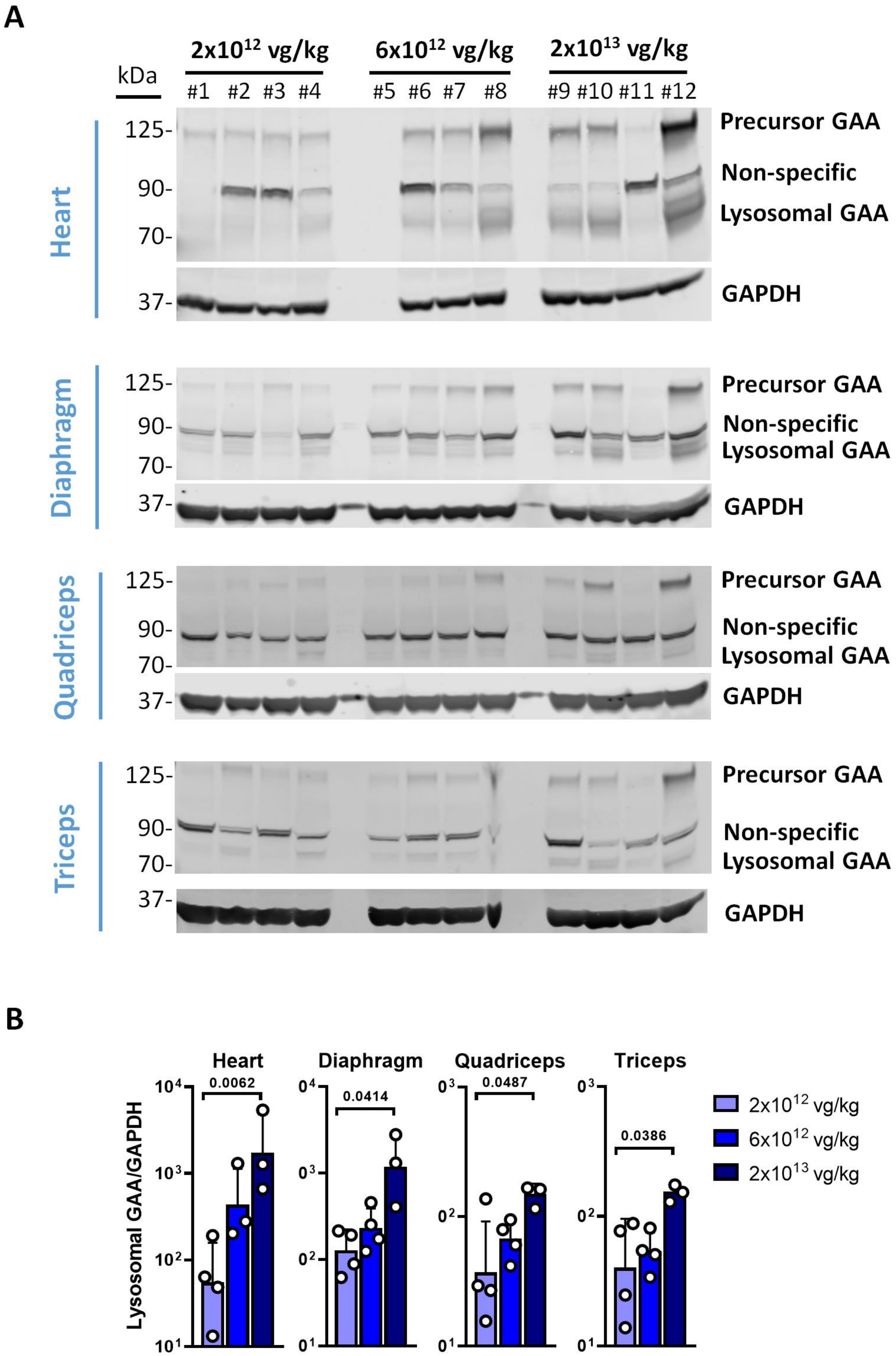

Supplementary Fig. 7. GAA antigen levels in muscles from NHPs. **A** Western blot analysis of NHPs muscle lysates with an anti-human GAA antibody 28 days after the administration of SPK-3006 vector at the indicated doses. **B** Quantification of lysosomal GAA protein bands from the corresponding Western blots depicted in (A) and normalized by GAPDH (data shown as average ± SD). Statistical analysis: One-way ANOVA; *n* = 4 animals for the $2x10^{12}$ vg/kg and $6x10^{12}$ cohorts, n = 3 animals for the $2x10^{13}$ vg/kg cohort (#11 was excluded from the analysis).

Supplementary Figure 8

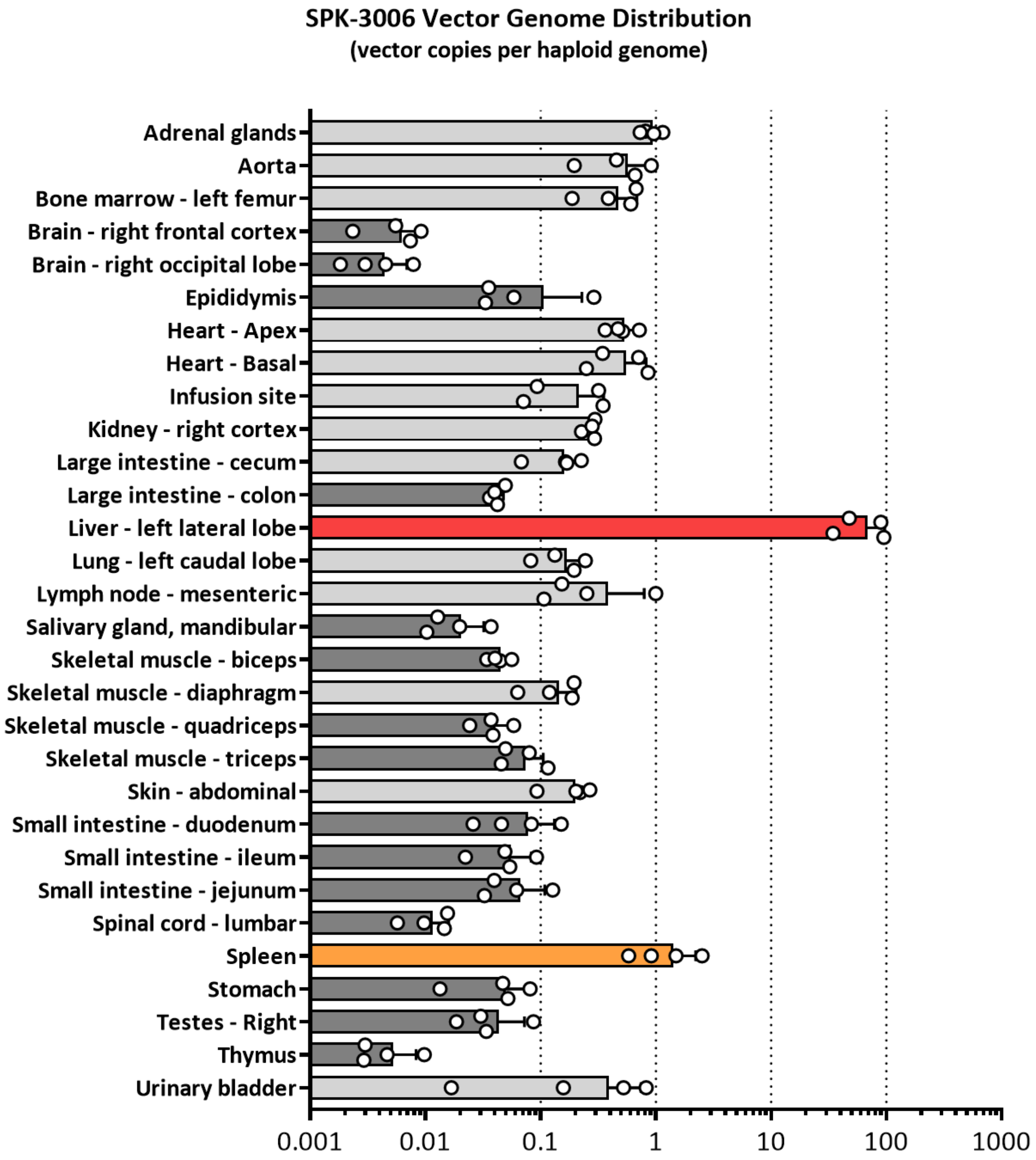

Supplementary Fig. 8. Vector genome biodistribution in tissues from NHPs. Vector copies per haploid genome in tissue isolates from NHPs administered with $2x10^{13}$ vg/kg of SPK-3006. The red bar indicates ≥ 30 copies; the orange bar indicates ≥ 1 copies; light

grey bars indicate ≥ 0.1 copies; dark grey bars indicate ≤ 0.1 copies. Values are presented as average ± SD (n = 4 animals).

Supplementary Tables

Supplementary Table 1. hGAA antigen levels and IgG anti-hGAA in plasma from NHPs dosed with SPK-3006

GAA Antigen in Plasma (Total fluorescence)						Anti-hGAA IgG (µg/ml)
**SPK-3006**	**ID**	**Day 7**	**Day 14**	**Day 21**	**Day 28**	**Day 28**
$2x10^{12}$ vg/kg	1	2762	1329	665	N.D.	N.D.
	2	1448	2017	1309	1255	0.060
	3	752	1153	1231	1265	0.116
	4	1543	1878	1918	1350	0.148
$6x10^{12}$ vg/kg	5	1992	3870	4131	2998	0.156
	6	2497	4766	4409	2611	1.661
	7	1713	2097	2041	2272	0.120
	8	4821	7486	9872	9748	0.190
$2x10^{13}$ vg/kg	9	2561	7814	7802	7309	0.070
	10	2326	13542	8617	6293	2.096
	11	951	1861	2427	799	0.935
	12	4854	13087	3894	2389	1.295

Supplementary Table 2. Clinical chemistry parameters in NHPs dosed with SPK-3006

ID	Timepoint (days)	AST (U/L)	ALT (U/L)	Creatine Kinase (U/L)	Creatinine (mg/dL)	Triglyceride (mg/dL)	Glucose (mg/dL)	C-reactive protein	Weight (Kg)
1	Baseline	41	32	3191	0.8	62	125	1.5	5.2
	7	80	38	5038	0.7	52	98	3.1	5.3
	14	40	39	140	0.6	46	86	1.2	5.6
	21	28	24	176	0.7	75	94	0.6	5.6
	28	N.D.	N.D.	N.D.	N.D.	N.D.	N.D.	N.D.	5.7
2	Baseline	23	20	150	0.7	38	89	0.8	4.7
	7	38	24	480	0.7	52	60	2	5.0
	14	34	26	213	0.8	49	88	1.4	5.1
	21	25	18	153	0.8	42	100	0.7	5.1
	28	27	27	153	0.8	50	102	0.9	5.2
3	Baseline	45	68	190	1	28	101	0.2	5.5
	7	49	52	238	0.8	66	67	1.6	5.7
	14	44	49	127	0.9	51	90	0.4	5.8
	21	44	42	979	0.9	49	111	0.5	5.8
	28	45	48	625	1	36	145	0.7	5.8
4	Baseline	40	31	1002	0.9	57	124	0.3	5.1
	7	46	27	365	0.9	51	88	2.4	5.3
	14	43	32	250	0.9	65	110	0.3	5.6
	21	48	34	1163	0.8	49	99	0.5	5.5
	28	45	33	358	0.9	68	138	0.7	5.6
5	Baseline	63	98	1008	1	35	136	0.1	3.6
	7	49	61	475	0.9	30	134	0.2	3.5
	14	43	46	176	0.9	55	91	1.3	3.8
	21	55	46	906	0.8	49	92	0.3	3.7
	28	47	44	170	0.9	40	136	1	3.8
6	Baseline	31	50	323	0.9	94	90	0.8	5.2
	7	32	34	338	0.9	71	82	1.2	5.3
	14	38	35	129	0.8	78	63	1.5	5.6
	21	33	31	214	0.8	72	87	1.3	5.8
	28	39	41	153	0.9	59	96	2.5	5.7
7	Baseline	42	42	326	0.8	50	73	1	4.9
	7	37	33	217	0.7	100	56	0.5	4.8
	14	36	31	129	0.7	84	68	1	5.1
	21	28	24	411	0.7	57	80	0.5	5.2
	28	40	31	1243	0.7	65	106	2.1	5.2
8	Baseline	51	47	3386	0.9	59	88	1.5	6.0
	7	37	57	236	0.8	74	57	4.2	6.2

	14	34	34	189	0.8	54	78	0.9	6.5
	21	30	30	192	0.9	60	67	1	6.5
	28	32	36	183	1	52	87	2.4	6.4
9	Baseline	25	36	223	0.7	53	91	0.2	5.0
	7	29	28	165	0.7	60	83	1	5.1
	14	35	32	314	0.6	55	68	0.4	5.3
	21	25	31	214	0.7	76	88	0.2	5.3
	28	27	29	147	0.8	42	96	0.3	5.4
10	Baseline	34	43	371	0.9	60	105	0.5	4.2
	7	48	36	454	1	65	104	0.9	4.4
	14	40	35	372	0.8	64	92	0.8	4.6
	21	35	31	680	0.8	49	108	0.6	4.6
	28	197	138	1531	1.1	62	161	9.8	4.8
11	Baseline	28	36	157	0.7	67	83	0.9	4.5
	7	33	33	150	0.8	65	66	1.2	4.6
	14	34	29	232	0.8	65	69	1	4.7
	21	33	28	240	0.8	64	76	1.2	4.7
	28	38	52	188	0.8	69	91	3	4.9
12	Baseline	24	29	83	0.8	52	102	1.3	5.3
	7	34	47	409	0.9	48	83	1.4	5.2
	14	31	43	77	0.7	62	82	2.1	5.4
	21	27	36	115	0.9	53	114	2.1	5.7
	28	30	34	109	0.8	42	93	1.6	5.5

Supplementary Table 3. List of primers/probes used in the study

Gene	Primer/Probe	Sequence
Human GAA cDNA	Forward	5’-AGATACGCCGGACATTGGACTG-3
	Reverse	5’-GCACGCCCAGCAGATTGAAC-3’
	Probe	5’-GTGTGGTCCTCTTGGGAGC-3’
Titin gene	Forward	5’-AGAGGTAGTATTGAAAACGAGCGG-3’
	Reverse	5’-GCTAGCGCTCCCGCTGCTGAAGCTG-3’
	Probe	5’-TGCAAGGAAGCTTCTCGTCTCAGTC-3’
Mouse *Gaa* (*Gaa-/-* genotyping)	Forward (mutated allele)	5’-CGTTGGCTACCCGTGATATT-3’
	Forward (wild-type allele)	5’-TCCTGAGCCCAAACACTTCT-3’
	Reverse	5’-TCCTGAGCCCAAACACTTCT-3’
Mouse *Cd4* (*Cd4-/-* genotyping)	Forward (mutated allele)	5’-GTGTTGGGTCGTTTGTTCG-3’
	Forward (wild-type allele)	5’-CCTCTTGGTTAATGGGGGAT-3’
	Reverse	5’-TTTTTCTGGTCCAGGGTCAC-3’
