# Supplementary material for: Hepatic expression of GAA results in enhanced enzyme bioavailability in mice and non-human primates
Source: Nat Commun. 2021 Nov 4;12:6393. doi: 10.1038/s41467-021-26744-4 (PMC8568898; doi:10.1038/s41467-021-26744-4)
Supplement: Supplementary file 2 — Reporting Summary [file 41467_2021_26744_MOESM2_ESM.pdf]

nature portfolio

Corresponding author(s): Federico Mingozzi

Last updated by author(s): Aug 31, 2021

Reporting Summary

Nature Portfolio wishes to improve the reproducibility of the work that we publish. This form provides structure for consistency and transparency in reporting. For further information on Nature Portfolio policies, see our Editorial Policies and the Editorial Policy Checklist.

Statistics

For all statistical analyses, confirm that the following items are present in the figure legend, table legend, main text, or Methods section.

n/a	Confirmed	
☐	☒	The exact sample size (n) for each experimental group/condition, given as a discrete number and unit of measurement
☐	☒	A statement on whether measurements were taken from distinct samples or whether the same sample was measured repeatedly
☐	☒	The statistical test(s) used AND whether they are one- or two-sided<br>*Only common tests should be described solely by name; describe more complex techniques in the Methods section.*
☒	☐	A description of all covariates tested
☐	☒	A description of any assumptions or corrections, such as tests of normality and adjustment for multiple comparisons
☐	☒	A full description of the statistical parameters including central tendency (e.g. means) or other basic estimates (e.g. regression coefficient) AND variation (e.g. standard deviation) or associated estimates of uncertainty (e.g. confidence intervals)
☐	☒	For null hypothesis testing, the test statistic (e.g. F, t, r) with confidence intervals, effect sizes, degrees of freedom and P value noted<br>*Give P values as exact values whenever suitable.*
☒	☐	For Bayesian analysis, information on the choice of priors and Markov chain Monte Carlo settings
☒	☐	For hierarchical and complex designs, identification of the appropriate level for tests and full reporting of outcomes
☒	☐	Estimates of effect sizes (e.g. Cohen's d, Pearson's r), indicating how they were calculated

Our web collection on statistics for biologists contains articles on many of the points above.

Software and code

Policy information about availability of computer code

Data collection	Densitometry analysis was conducted using Compass® software (Bio-Techne) version 5.0.1
Data analysis	All data were analyzed using GraphPad Prism 6; Images were analyzed ImageJ software version 1.51

For manuscripts utilizing custom algorithms or software that are central to the research but not yet described in published literature, software must be made available to editors and reviewers. We strongly encourage code deposition in a community repository (e.g. GitHub). See the Nature Portfolio guidelines for submitting code & software for further information.

Data

Policy information about availability of data

All manuscripts must include a data availability statement. This statement should provide the following information, where applicable:
- Accession codes, unique identifiers, or web links for publicly available datasets
- A description of any restrictions on data availability
- For clinical datasets or third party data, please ensure that the statement adheres to our policy

All data presented in the manuscript are available in the Source Data file.

Field-specific reporting

Life sciences study design

All studies must disclose on these points even when the disclosure is negative.

Sample size	Sample size was defined based on the previous experience with mouse models of Pompe disease (Puzzo et al., Science Translational Medicine 2017; Colella et al., EBiomedicine 2020; Cagin et al., Molecular Therapy 2020). The Gaa-/- Cd4-/- mouse model used in this study appeared to have lower variability than the original immunocompetent Gaa-/- mouse. For monkey experiments, due to ethical concerns groups of 4 animals were used in each dose cohort, for a total of 12 animals.
Data exclusions	All animals were included in the analysis with the exception of the NHP study in which one animals from the high dose cohort (#11) was excluded from the statistical analyses due to unexplained lower levels of transgene expression not justified by the development of anti-hGAA IgG or by lower VGCN in liver. This has been clearly stated in the main text of the manuscript.
Replication	The number of sample units (n) represent the individual animals which are used for statistical analysis. Treatment groups were composed by different litters. Mouse experiments were repeated at least twice a yielded consistent results. The non-human primate experiment was performed once, although additional non-human primates studies with the same vector but different study design were performed and yielded the same results as the ones described in the manuscript.
Randomization	For mouse and non-human primate studies animals were randomly assigned to treatment cohorts.
Blinding	Operators in charge of sample analysis were blinded to the experimental design.

Reporting for specific materials, systems and methods

We require information from authors about some types of materials, experimental systems and methods used in many studies. Here, indicate whether each material, system or method listed is relevant to your study. If you are not sure if a list item applies to your research, read the appropriate section before selecting a response.

Materials & experimental systems

n/a	Involved in the study	
☐	☒	Antibodies
☒	☐	Eukaryotic cell lines
☒	☐	Palaeontology and archaeology
☐	☒	Animals and other organisms
☒	☐	Human research participants
☒	☐	Clinical data
☒	☐	Dual use research of concern

Methods

n/a	Involved in the study	
☒	☐	ChIP-seq
☒	☐	Flow cytometry
☒	☐	MRI-based neuroimaging

Antibodies

Antibodies used	Anti-human GAA (rabbit monoclonal, Abcam, cat. no. ab137068, clone EPR4716, dilution 1:1000); anti-mouse SQSTM1/p62 (mouse monoclonal, Abcam, cat. no. ab56416, clone 2C11, dilution 1:1000); anti-mouse PARK2/parkin (rabbit polyclonal, Proteintech, cat. no. 14060-1-AP, dilution 1:1000); anti-mouse GAPDH (mouse polyclonal, Thermo Fisher Scientific, cat. no. PA1-988, dilution 1:500); anti-mouse α-tubulin (mouse monoclonal, Sigma-Aldrich, cat. no. T9026, clone DM1A, 1:500); anti-mouse vinculin (mouse monoclonal, Sigma-Aldrich, cat. no. V9131, clone hVIN-1, dilution 1:250); anti-mouse laminin primary antibody (rabbit polyclonal; Dako, Agilent, cat. no. Z0097, dilution 1:400).
Validation	Anti-human GAA, immunogen: human GAA. Protocol validated for Western blot and WES in tissues and plasma from mice and non-human primates by testing multiple dilutions of the antibody (Puzzo et al., Sci Transl Med 2017; Colella et al., EBiomedicine 2020; Cagin et al., Mol Ther 2020);<br>Anti-mouse SQSTM1/p62, protocol validated by testing multiple dilutions of the antibody in mouse tissues (Puzzo et al., Sci Transl Med 2017; Colella et al., EBiomedicine 2020; Cagin et al., Mol Ther 2020, see also manufacturer's web site);<br>Anti-mouse PARK2/parkin, protocol validated by testing multiple dilutions of the antibody in mouse tissues (Colella et al., EBiomedicine 2020, see also manufacturer's web site);<br>Anti-mouse GAPDH, protocol validated by testing multiple dilutions of the antibody in mouse tissues (see also manufacturer's web site);<br>Anti-mouse α-tubulin, protocol validated by testing multiple dilutions of the antibody in mouse tissues (see also manufacturer's web site);<br>Anti-mouse vinculin, protocol validated by testing multiple dilutions of the antibody in mouse tissues (see also manufacturer's web site);<br>Anti-mouse laminin primary antibody, protocol validated by testing multiple dilutions of the antibody in mouse tissues (see also manufacturer's web site).

Animals and other organisms

Policy information about studies involving animals; ARRIVE guidelines recommended for reporting animal research

Laboratory animals	A mouse colony of Gaa-/- Cd4-/- double knock out mice was generated for this study by breeding Gaa-/- mice (B6;129-Gaatm1Rabn/J stock no. 004154) with Cd4-/- mice (B6.129S2-Cd4tm1Mak/J stock no. 002663), both from Jackson Laboratories. For all studies comparing ERT with AAV gene transfer, male Gaa-/-Cd4-/- and healthy littermates of 2 months of age were used. For the chaperone study, male Gaa-/-Cd4-/- and healthy littermates of 4 months of age were used. To compare SPK-3006 with AAV8-hAAT-sp7-Δ8-coGAA 8-10 weeks old male C57BL/6 mice were used. For the non-human primate experiment, male Macaca mulatta animals aged 3-5 years were used.
Wild animals	No wild animals were used in the study.
Field-collected samples	No field collected samples were used in the study.
Ethics oversight	Mouse experiments were performed according to the French and European legislation on animal care and experimentation (2010/63/EU) and approved by Genethon's ethical committee (protocol no. 2017-011-B #13643).<br>For the studies in non-human primates, the in vivo procedures were performed at a Charles River's facility (Mattawan, MI, United States), according to the Animal Welfare Act (Title 7 United States Code, Sections 2131-2159) and Animal Welfare Regulations (Title 9 Code of Federal Regulation, Parts 1, 2 and 3). the study was approved by the Institutional Care and Use Committee (IACUC) of Charles River Mattawan (protocol no. 2377-015, approved on April 23, 2018).

Note that full information on the approval of the study protocol must also be provided in the manuscript.
